# Accuracy Meets Feasibility for the Structures and Rotational Constants of the Molecular Bricks of Life: A Joint Venture of DFT and Wave-Function Methods

**DOI:** 10.1021/acs.jpclett.3c01380

**Published:** 2023-06-21

**Authors:** Vincenzo Barone

**Affiliations:** Scuola Normale Superiore di Pisa, Piazza dei Cavalieri 7, 56126 Pisa, Italy

## Abstract

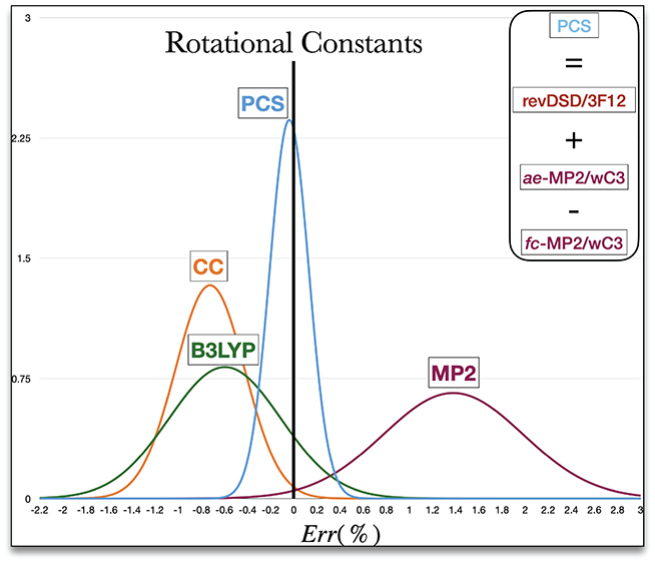

A fully unsupervised computational protocol is proposed with the aim of obtaining reliable structural properties for molecular bricks of life in the gas phase. The results of the new composite scheme approach spectroscopic accuracy at a moderate cost without any empirical parameter in addition to those of the underlying electronic structure method. The whole workflow is fully automated and provides optimized geometries and equilibrium rotational constants. Direct comparison with experimental ground state rotational constants can be performed thanks to the effective computation of vibrational corrections in the framework of second-order vibrational perturbation theory. The results for all the nucleic acid bases and several flexible molecules of biological or medicinal interest show that the accuracy of the new tool is close to that delivered by state-of-the-art composite wave function methods for small semirigid molecules.

The experimental study of biomolecule building blocks in the gas phase has recently attracted increasing attention owing to the development of spectrometers coupling supersonic-jet expansion^1^ with laser ablation.^2^ This has allowed the application of high-resolution spectroscopy to the main molecular bricks of life (e.g., amino acids, peptides, sugars, nucleobases, and even nucleosides), which are usually thermolabile molecules with high melting points. However, a direct interpretation of the spectroscopic outcome in terms of structural and dynamic features at the molecular level is not straightforward. In particular, high resolution spectra, while conveying a wealth of information, are very congested so that their assignment and interpretation become very difficult. In this respect, quantum chemical (QC) computations can play an invaluable role, especially because gas phase is their most natural playground.^3^ Unfortunately, state-of-the-art QC approaches, which have reached an impressive accuracy for small semirigid molecules,^4^ are characterized by a very unfavorable scaling with the dimension of the system under investigation. Therefore, the first challenge for accurate computational studies is related to the dimensions of biomolecule building blocks, which contain a few dozen atoms. While the recent development of low-scaling accurate methods, especially in connection with explicitly correlated (F12) models (see e.g., refs (5 and 6). and references therein), allows the computation of accurate electronic energies, the situation is different for geometries (of interest especially in microwave spectroscopy) and vibrational frequencies (of interest for thermochemistry, kinetics, and, together with transition moments, for IR and Raman spectroscopy). A second challenge is related to the large number of low-energy structures (conformers, but also tautomers or other isomers) and to the fast relaxation of some of them to more stable counterparts due to the presence of low interconversion energy barriers.^7^ In this respect, besides the increase of computational effort, the major problem is related to the inefficiency of the powerful local optimization techniques developed for semirigid molecules in the case of flexible systems, which require the exploration of rugged potential energy surfaces (PESs).^8^ For the reasons mentioned above, reliable theoretical support for current high-resolution spectroscopy studies requires an integrated strategy that employs QC models of increasing accuracy in the different phases of an exploration/exploitation workflow. The main steps of this strategy^8,9^ can be summarized as follows:

1. Unsupervised perception of the molecular system to identify hard and soft degrees of freedom,^10^ fast exploration of soft degrees of freedom^8^ and analysis of relaxation paths between pairs of adjacent energy minima.^11^
2. Determination of accurate geometries and force fields for the most stable structures not involved in fast relaxation paths.^8^
3. Evaluation of accurate electronic energies and properties for the final panel of low-energy minima.^12^
4. Computation of relative populations and spectroscopic parameters at the temperature of interest using the quantities obtained in steps 2 and 3.^13^

Satisfactory solutions for steps 1, 3, and, at least partially, 4 have been proposed over the last years.^6,14,15^ Therefore, the focus of the present work is on step 2. From an experimental point of view, extremely accurate structural data in the gas phase can be obtained from microwave (MW) spectra. However, the experimental rotational constants for the ground vibrational state (B_i_^0^) include vibrational corrections (ΔB_i_^vib^) in addition to equilibrium values (B_i_^eq^).^16^ Since the experimental determination of vibrational corrections is practically impossible except for very small molecules, their computation by QC methods (leading to the so-called semi experimental (SE) equilibrium rotational constants, B_i_^SE^) has emerged as a very accurate alternative.^4,17^ Actually, the contribution of vibrational corrections is typically well below 1% of the corresponding equilibrium rotational constant, so that they can be safely determined at affordable levels of theory.^18,19^ For small molecules, isotopic substitutions produce a sufficient number of different SE equilibrium rotational constants to allow the determination of very accurate SE equilibrium geometries by a nonlinear least-squares fitting.^20^ This approach cannot be pursued for larger molecules due to the lack of experimental data, but the quality of different QC methods can be estimated from the direct comparison between the SE rotational constants of the main isotopologue and their counterparts issued from QC geometry optimizations. This is the route followed in the present contribution for validating a new QC approach, which will be termed the Pisa Composite Scheme (PCS).

The dimensions of the target systems restrict the sophistication of suitable methods to MP2 and density functionals, for which analytical gradients and Hessians can be computed with reasonable computer resources (see, e.g., ref (21) and references therein). As a matter of fact, these are the levels of theory routinely employed to aid experimental spectroscopic studies.^22,23^ However, in my opinion, the possibilities offered by modern models (e.g., double hybrid functionals) have not yet been fully exploited. Based on these premises, in previous works we employed with remarkable success the rev-DSD-PBEP86-D3BJ functional^24^ (hereafter rDSD) in conjunction with a partially augmented triple-ζ basis set (jun-cc-pVTZ,^25^ hereafter j3).^26,27^ Noted is that, while the D4 model for empirical dispersion^28^ might deliver marginally better results for energies,^24^ D3BJ and D4 contributions to intramolecular geometrical parameters are virtually indistinguishable, and above all, analytical second derivatives are available for the D3BJ version.^21^ The systematic nature of the errors permits to improve significantly the rDSD/j3 geometrical parameters by a linear regression (LR) approach.^29^ Even better results can be obtained resorting, when possible, to templating molecules (TMs) sharing structural similarities with the species under study and whose accurate equilibrium structure is already available.^29^ The resulting model (referred to as the nano-LEGO^29^ or LEGO-Bricks^30^ approach) has met considerable success, but suffers from two main problems. From the one side, the use of empirical parameters in the LR approach is not fully satisfactory, and from the other side, the number of available accurate structures for the fragments to be employed as TMs is limited. For instance, while almost all of the amino acids (a notable exception being tryptophan) can be built from available fragments, the situation is different for nucleobases (especially when taking into account different tautomeric forms) and, above all, for the varied scaffolds of drugs. Therefore, it would be worthwhile to devise a parameter-free approach able to deliver accurate geometrical structures at an affordable cost for medium-sized molecular systems.

It is well-known that converged results can be obtained by double-hybrid functionals only using at least (partially) augmented quadruple-ζ basis sets (see, e.g., ref (31) and references therein). Starting from this level, several test computations showed that g and diffuse d, f functions on second- and third-row atoms together with f and diffuse functions on first-row atoms produce negligible changes in the geometrical parameters with a considerable reduction of the computational cost. As a matter of fact, the cc-pVTZ-F12 basis set^32^ (hereafter 3F12), purposely developed for explicitly correlated computations, offers a nearly optimal cost/accuracy compromise also in conjunction with the rDSD functional. This combination of the functional and basis set (rDSD/3F12) is taken here as the new standard for geometry optimizations. However, at these levels of accuracy, the core-valence (CV) correlation cannot be neglected. Therefore, adopting the same recipe employed in the “cheap” family of composite methods,^33,34^ CV contributions can be evaluated effectively from the difference between all electron (ae) and frozen core (fc) MP2 computations performed in conjunction with the cc-pwCVTZ basis set^35^ (hereafter wC3). It is known that this basis set slightly underestimates the core correlation,^36^ but the error is generally smaller than 0.001 Å. Furthermore, the MP2 method slightly overestimates the core correlation;^37^ thus, there is a partial compensation of errors, with the final error being much smaller than 0.001 Å (see, for instance, Table S4 of ref (38)). Optimized geometries including CV correlation can be obtained without any modification of standard electronic structure codes by the so-called “geometry” scheme,^39,40^ which is based on the assumption that the additivity approximation can be directly applied to geometrical parameters. In this framework, the results of three separate geometry optimizations (which can be performed independently and possibly on different nodes) are combined together. Since the size of the wC3 or 3F12 basis sets and the cost of MP2 or rDSD computations are comparable (actually slightly lower in the former case), the inclusion of CV correlation can formally increase the needed resources (computer nodes or elapsed time on a single node) up to a factor of 3. However, it will be shown that this increase is accompanied by an unquestionable improvement. Actually, the accuracy of the PCS geometries suggests their systematic use in the computation of high-quality electronic energies by state-of-the-art composite wave function methods. In this way, computation of vibrational corrections, geometry optimizations, and single point evaluation of electronic energies require comparable resources (time and memory) for the molecular sizes (a few dozen atoms belonging to the first three rows of the periodic table) of interest in the present context. Furthermore, the computation of all of the needed pieces of information can be fully automatized with the aid of widely employed electronic structure codes. In particular, all the computations reported in the present paper have been performed by the Gaussian package^41^ employing simple scripts.

Since, as already mentioned, nearly all amino acids can be effectively built from available templating molecules, and have been recently investigated in detail,^11,42^ the case studies chosen to validate the PCS results are the nucleobases and flexible drugs shown in Figures 1 and 2. Table 1 reports the rotational constants computed at different levels of theory for all of the standard nucleobases (in their keto-amine forms) and 2-thiouracil, which has been included to show that the methodology can also be applied to molecules containing third-row atoms. Furthermore, in the case of cytosine, also the low-energy enol–amine (EA) and keto–imine (KI) tautomers detected in the gas phase have been considered.^43^ Based on previous experience,^18^ the vibrational corrections needed to obtain SE equilibrium rotational constants have been computed at the B3LYP-D3BJ/6–31+G* level (hereafter B3/SVP).

**Figure 1:**
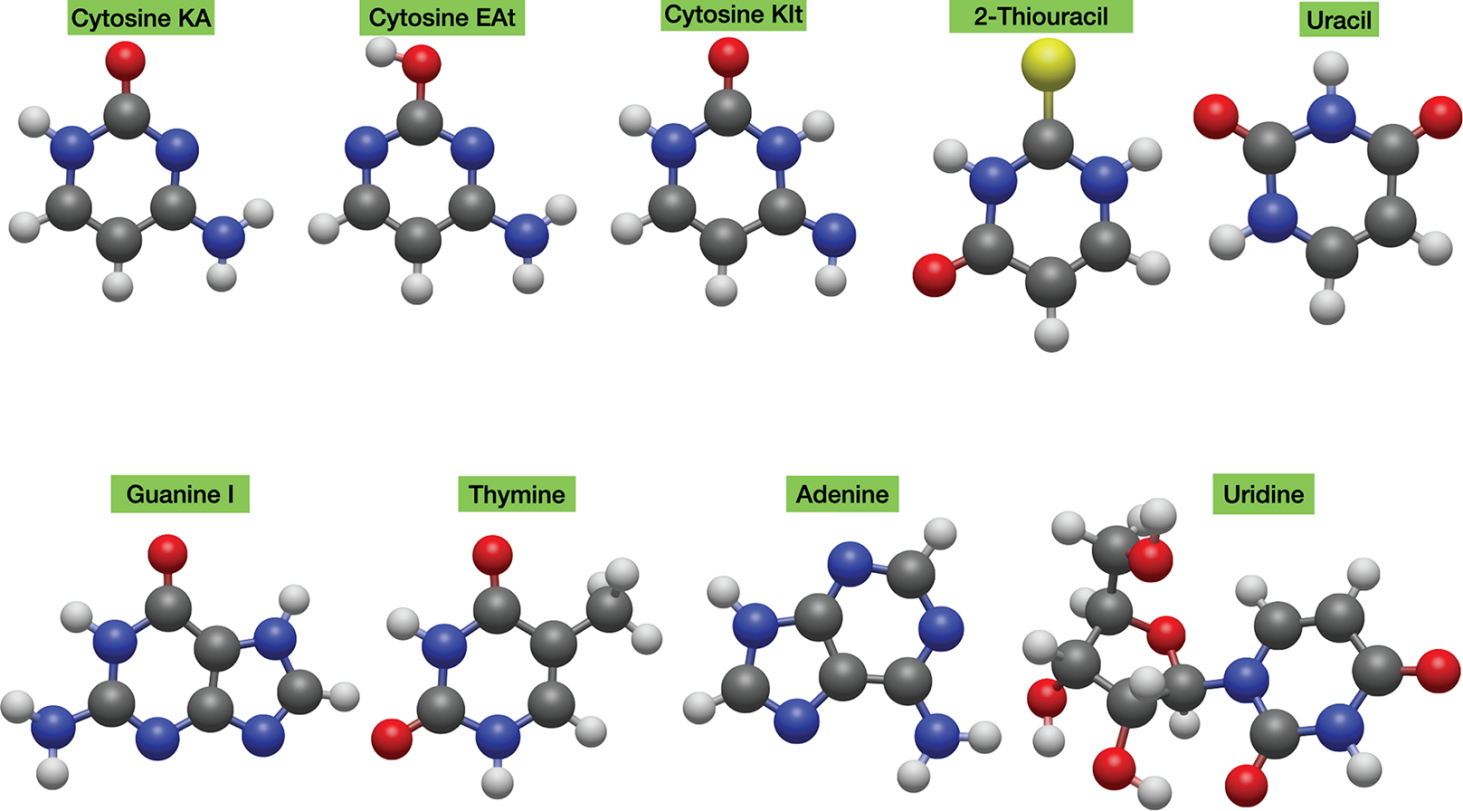
Structures of the nucleobases and uridine nucleoside.

**Figure 2:**
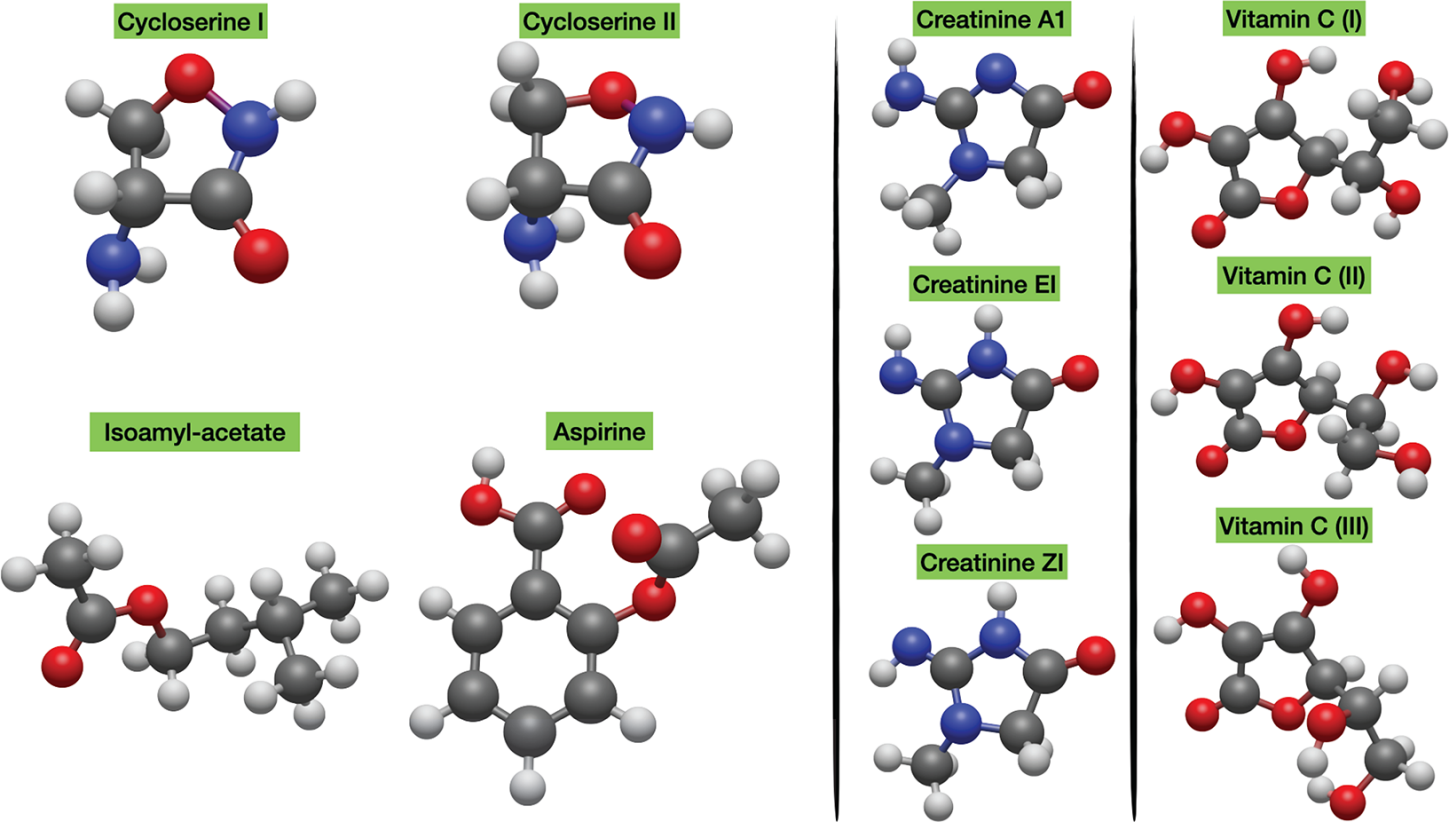
Structures of the flexible drugs selected to build the ROT30 database.

**Table 1:** Equilibrium Rotational Constants (*B*_*i*_^eq^) and Vibrational Corrections (Δ*B*_*i*_^vib^) for the Canonical Forms of Nucleobases, the Other Two Stable Tautomers of Cytosine (EA and KI), and the Uridine Nucleoside Obtained by Different Methods

axis	Δ*B*_*i*_^vib^(B3/SVP)	*B*_*i*_^eq^(SE)^a^	*B*_*i*_^eq^(PCS)	*B*_*i*_^eq^(B3/SVP)	*B*_i_^*e*q^(CC)^b^
uracil					
a	–27.6	3911.5	3914.3	3871.2	3861.8
b	–10.6	2034.3	2033.3	2006.6	2004.1
c	–7.4	1338.3	1338.2	1321.6	1319.4
2-thiouracil					
a	–22.8	3578.0	3582.8	3541.9	3550.5
b	–6.4	1321.3	1321.7	1300.6	1306.6
c	–4.9	964.9	965.5	951.3	955.1
thymine					
a	–23.4	3224.6	3224.9	3188.2	3184.2
b	–7.7	1412.5	1412.4	1394.7	1392.0
c	–5.6	988.2	988.2	976.1	974.4
adenine					
a	–13.7	2385.6	2390.7	2367.0	2352.7
b	–8.7	1582.1	1582.8	1562.2	1563.6
c	–5.8	952.1	952.5	941.2	939.9
guanine					
a	–11.6	1933.8	1934.1	1910.4	
b	–6.7	1128.4	1127.9	1115.1	
c	–4.2	713.2	713.0	704.7	
cytosine					
a	–27.4	3899.0	3904.3	3850.9	3842.9
b	–9.4	2034.4	2035.9	1992.1	2009.0
c	–7.9	1338.2	1338.5	1312.9	1320.6
cytosine (EA)					
a	–27.6	3979.5	3984.7	3935.4	3928.5
b	–11.0	2020.0	2020.9	1996.1	1993.2
c	–8.6	1341.1	1341.5	1324.9	1323.7
cytosine (KI)					
a	–29.4	3877.6	3881.8	3830.0	3841.0
b	–11.0	2037.3	2036.2	2010.4	1994.7
c	–7.7	1335.7	1335.6	1318.4	1312.9
uridine					
a	–9.0	895.0	892.8	881.9	
b	–2.9	338.5	338.3	334.4	
c	–2.5	272.6	272.2	268.7	

a SE equilibrium rotational constants obtained from the experimental ground state rotational constants and vibrational corrections computed at the B3/SVP level. Experimental data are from refs (34 and 43−48) for uracil, 2-thiouracil, thymine, adenine, guanine, cytosine, and uridine, respectively. All of the values are given in MHz.

b CCSD(T)/cc-pVTZ(-*f*) from ref (49). except for 2-thiouracil, CCSD(T) from ref (34).

The results show unambiguously that the B3LYP and MP2 methods routinely employed in the interpretation of MW spectra can provide, at most, qualitative trends: indeed, at these levels the computation of vibrational corrections is not warranted. Already rDSD/j3 computations perform a respectable job, and extension of the basis set together with inclusion of CV correlation (which plays a comparable role) leads to quantitative agreement with the SE equilibrium rotational constants.

As a matter of fact, the PCS errors are smaller by about 1 order of magnitude than those delivered by standard DFT, MP2, and CCSD(T)/cc-pVTZ(-*f*)^49^ approaches and fulfill the goal of mean unsigned errors below 0.1%, which is considered the gold standard of the most accurate computations even for small semirigid molecules.^4,50^ Since the SE equilibrium structure of uracil is available, a direct comparison of geometrical parameters is also possible, which shows that the mean unsigned error of bond lengths is reduced from 0.003 to 0.002 and 0.001 Å (the corresponding maximum errors being 0.005, 0.003, and 0.002 Å, respectively) when going from rDSD/j3 to rDSD/3F12 and PCS levels. At the same time, the valence angles are always sufficiently accurate. It is especially worth mentioning that rotational constants (hence geometrical parameters) of remarkable accuracy (0.2%) can be obtained by the PCS model for uridine, a flexible molecule containing 29 atoms, which is beyond the range of application of state-of-the-art QC methods.

Together with covalent interactions, most molecules of current biological and medicinal interest are quite flexible and involve significant noncovalent (e.g., hydrogen-bond) interactions. Both features increase the difficulty of predicting accurate geometrical parameters due to the need to describe correctly dispersion interactions and to the possible presence of strong vibrational averaging effects in the experimental data. As a consequence, it is particularly important to analyze the performance of the new composite scheme for a panel of typical medium-sized organic molecules of pharmaceutical interest with various functional groups and some structural flexibility. The reference experimental data are usually accompanied by the results of standard QC calculations in order to assign measured MW signals, with this allowing for additional comparisons. The rotational constants of the ten molecules shown in Figure 2 have been chosen to build the new ROT30 compilation (see Table 2), which is intended to supersede the ROT25 set.^51^ Actually, three molecules (isoamyl acetate, aspirin, and conformer III of vitamin C) were already present in the ROT25 compilation, with this allowing the researchers to appreciate the accuracy gain offered by the new model. Thanks to the efficiency of the vibrational second-order perturbation theory (VPT2) engine implemented in the Gaussian software,^52,53^ all the vibrational corrections are now computed at the B3/SVP level, which delivers results significantly more accurate than the Hartree–Fock method in conjunction with a double-ζ basis set (HF/DZ) employed in ref (51). Inspection of Table 2 shows that while the trends are roughly the same, the HF/DZ errors are often outside the error bar of the PCS results especially for some *B*_*a*_ constants. In more general terms, the PCS results represent a significant improvement over the current standards in MW studies of medium-sized molecules.^22,23^ In fact, the rDSD/3F12 errors are already smaller than those delivered by the B2PLYP-D3 functional in conjunction with a quadruple-ζ basis set, which was considered extremely accurate, but not generally exploitable in ref (51). Then, inclusion of CV correlation further improves the situation, reaching a quality comparable with that of state-of-the-art composite methods for small semirigid systems.

**Table 2:** Equilibrium Rotational Constants (*B*_*i*_^eq^) and Vibrational Corrections (Δ*B*_*i*_^vib^) for Flexible Drugs

axis	Δ*B*_*i*_^vib^(B3/SVP)^a^	*B*_*i*_^eq^(SE)^b^	*B*_*i*_^eq^(PCS)	*B*_*i*_^eq^(B3/SVP)	*B*_*i*_^eq^(rDSD/3F12)
isoamyl acetate					
a	–28.9 (−41.6)	3309.8	3313.9	3271.1	3301.4
b	–8.6 (−6.8)	721.6	718.4	710.1	715.8
c	–8.0 (−7.9)	698.1	696.7	687.7	694.1
vitamin C (I)					
a	–14.4	1576.9	1575.9	1552.0	1569.9
b	–5.7	720.9	720.2	711.9	717.8
c	–4.3	528.6	528.1	520.8	526.2
vitamin C (II)					
a	–21.1	1493.8	1492.2	1485.4	1487.1
b	–3.5	681.6	680.2	669.9	677.7
c	–4.0	579.0	578.2	568.4	576.0
vitamin C (III)					
a	–8.8 (−8.5)	1464.5	1463.6	1439.7	1458.1
b	–8.4 (−7.7)	768.9	766.5	763.8	763.8
c	–5.9 (−5.2)	575.4	579.5	576.5	577.4
aspirin					
a	–7.6 (−10.2)	1163.7	1165.5	1156.1	1160.9
b	–4.7 (−5.0)	767.3	766.1	759.0	763.4
c	–3.3 (−4.0)	512.3	512.1	507.5	510.2
cycloserine I					
a	–19.0	3704.0	3700.4	3633.4	3684.5
b	–28.3	3188.8	3191.1	3155.8	3181.1
c	–15.6	1831.1	1829.5	1802.5	1823.5
cycloserine II					
a	–38.6	3687.6	3684.6	3607.6	3669.8
b	–22.4	3191.0	3191.8	3161.2	3182.4
c	–19.6	2013.3	2009.3	1963.3	2001.7
creatinine ZI					
a	–38.4	3880.8	3866.4	3802.2	3852.7
b	–11.5	1836.9	1835.2	1813.5	1829.5
c	–10.4	1271.4	1269.6	1251.6	1265.7
creatinine EI					
a	–41.1	3931.3	3920.6	3870.8	3909.8
b	–11.9	1822.2	1822.3	1797.5	1816.1
c	–11.2	1269.3	1267.6	1249.6	1263.8
creatinine A1					
a	–29.3	3870.2	3859.6	3801.7	3845.6
b	–14.0	1845.5	1844.0	1820.1	1838.4
c	–11.9	1278.8	1277.3	1258.8	1273.8

a In parentheses, the HF/DZ results from ref (51).

b SE equilibrium rotational constants obtained from the experimental ground state rotational constants and vibrational corrections computed at the B3/SVP level. Experimental data are from refs (54, 22, 23, 13, and 55) for isoamyl acetate, vitamin C, aspirin, cycloserine, and creatinine, respectively. All the values are given in MHz.

The relative deviations of different methods from the reference data are shown in Figure 3. It is apparent that vibrational corrections decrease the values of rotational constants, whereas CV correlation has just the opposite effect. Therefore, the tendency of B3LYP to overestimate bond lengths (hence underestimate rotational constants) explains the fair agreement between B3LYP equilibrium rotational constants and experimental ground state values. In the same vein, inclusion of CV correlation further worsens the already too large rotational constants obtained at the MP2 level. On the other hand, the balanced treatment of both contributions improves the already satisfactory rDSD/3F12 results. In any case, an unbiased analysis of the relationship between geometrical parameters and experimental rotational constants requires separate consideration of the CV correlation and vibrational corrections. The first contribution, which essentially shortens bond lengths, increases with the atomic number of the involved atoms: as a consequence, its effect is smaller for X–H than for X–Y bonds (with X, Y being second- or third-row atoms). Also the role of vibrational corrections is particularly important for bond lengths, but the leading term (related to cubic force constants) now produces ground state bond lengths longer than their equilibrium counterparts, with the X–H bonds being especially affected due to their strong anharmonicity.

**Figure 3:**
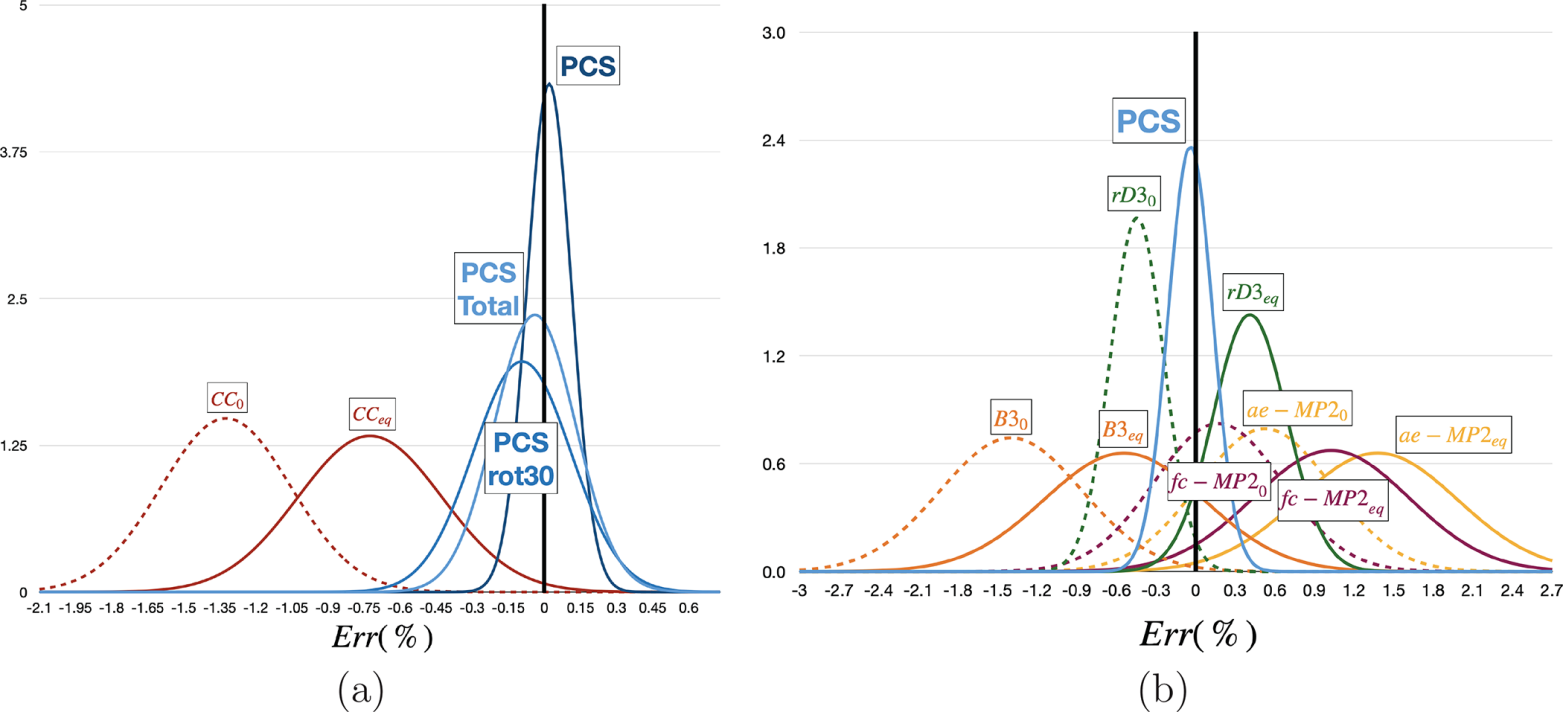
Relative (%) deviations of computed rotational constants from the reference experimental values for nucleobases (a) and the ROT30 set (b). The PCS relative deviations for the ROT30 and Total (i.e., ROT30 + nucleobases) sets are also included in panel a. Equilibrium and ground state values are denoted by “eq” and “0” subscripts, respectively. CC stands for CCSD(T)/ccpVTZ(-f), rD3 for rDSD/cc-pVTZ-F12, MP2 for MP2/cc-pwCVTZ, and B3 for B3LYP-D3BJ/6–31+G*.

All of these trends are well evidenced by the data collected in Figure 3. The relative mean unsigned error (RMUE) of rDSD results is 0.44% for the valence-only computations, −0.40% when adding only vibrational corrections, 0.49% when adding only CV correlation and 0.09% for the complete PCS model. It is also worth pointing out that the expected increase of the error when going from semirigid molecules (0.07% for the nucleobases) to flexible molecules involving noncovalent interactions (0.20% for the ROT30 set) is not huge. This last result is particularly relevant because only if covalent and noncovalent interactions are computed correctly can the “right answer for the right reason” be obtained, especially when environmental effects tune the results of intrinsic stereoelectronic effects (e.g., in large biomolecules or in condensed phase).

In order to show that the accuracy of the PCS results is not due to a fortuitous error compensation, a detailed comparison was performed with state-of-the-art results for the smallest molecule included in the ROT30 set (cycloserine). To this end, the equilibrium rotational constants of both low-energy structures of cycloserine have been computed by the very accurate explicitly correlated CCSD(T)-F12 method in conjunction with the cc-pVDZ-F12 basis set^56^ (hereafter this combination of method and basis set is labeled CC-F12). The results collected in Table 3 show that, at this level, the RMUE (0.42%) is about four times larger than its PCS counterpart (0.1%). However, inclusion of the same CV contributions employed in the PCS computations reduces the RMUE to a remarkable 0.04%. This result confirms that both vibrational corrections and CV correlation play a non-negligible role but can be computed with sufficient accuracy and reasonable cost at the B3/SVP and MP2/wC3 level, respectively. While PCS is slightly less accurate than CC-F12+CV, it is about 2 orders of magnitude less expensive and can be routinely applied to much larger systems (see, e.g., the uridine nucleoside in Table 1).

**Table 3:** Equilibrium Rotational Constants of Cycloserine (in MHz).

axis	SE^a^	PCS	CC-F12	CC-F12+CV
cycloserine I				
a	3704.0	3700.4	3686.0	3702.1
b	3188.8	3191.1	3176.2	3187.0
c	1831.1	1829.5	1823.7	1830.6
cycloserine II				
a	3687.7	3684.6	3670.2	3686.1
b	3191.0	3191.8	3179.7	3190.2
c	2013.3	2009.3	2004.8	2012.3

a The experimental ground state rotational constants are from ref (13) and the vibrational corrections from Table 2.

It is noteworthy that the CV corrections are entirely negligible for valence or dihedral angles, and their contribution to bond lengths mainly depends on the principal quantum numbers of the atoms involved in the bond. As a matter of fact, the bond length (*r*) between atoms *i* and *j* (with principal quantum numbers *n*_*i*_ and *n*_*j*_) is well approximated by the following recipe:
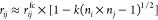
1where the frozen core *r*_*ij*_^fc^ value does not include the contribution of CV correlation and *k* is of the order of 1 mÅ. A further significant speedup can be obtained neglecting *d* functions on first-row atoms and reducing from 2 to 1 the number of *f* functions on second- and third-row atoms. Since all these simplifications have a negligible effect on the final accuracy of the results, the introduction of a single empirical parameter provides a low-cost (L) version of the composite scheme (LPCS), which will be fully described and validated in a forthcoming paper devoted to very large molecules. However, in the present context, the original PCS version has been systematically employed, since it has been possible to perform all the computations in reasonable times with the aid of a standard workstation.

In summary, a general strategy aimed at computing accurate geometries for molecules containing a few dozen atoms has been proposed. The new model (PCS) delivers highly reliable results with reasonable computer resources by means of a fully unsupervised workflow. In a broader context, a comprehensive compilation of PCS molecular structures (the PCS/23 database, which will be made available on our Web site) can integrate the SE127 compilation of very accurate semi experimental equilibrium geometries^57^ for the study of even larger molecules by the already mentioned nano-LEGO tool.^29^ At the same time, improved algorithms for the evaluation of vibrational corrections (which can become the rate determining step for proper comparison with MW rotational constants) would further extend the range of applications of the model. While work along these lines is in progress, already the present user-friendly implementation of the PCS tool paves the way toward its widespread application also by nonspecialists.

## Supporting Information Available

Transparent Peer Review report available (PDF)
