# Supplementary material for: Accuracy Meets Feasibility for the Structures and Rotational Constants of the Molecular Bricks of Life: A Joint Venture of DFT and Wave-Function Methods
Source: J Phys Chem Lett. 2023 Jun 21;14(25):5883–90. doi: 10.1021/acs.jpclett.3c01380 (PMC10316392; doi:10.1021/acs.jpclett.3c01380)
Supplement: Supplementary file 1 — jz3c01380_si_001.pdf [file jz3c01380_si_001.pdf]

jz-2023-013804.R1

Name: Peer Review Information for "Accuracy Meets Feasibility for the Structures and Rotational Constants of the Molecular Bricks of Life: a Joint Venture of DFT and Wave-Function Methods"

First Round of Reviewer Comments

Reviewer: 1

Comments to the Author

In this manuscript Barone propose a new strategy to predict and reproduce the structural properties of large molecular systems in gas phase. The proposed procedure works in the framework of DFT and do not use any kind of empirical parameter and need only the starting geometry. The reported large number of molecular tests, including nucleic acid and other important biological systems, demonstrate that the proposed method give accurate results comparable to the microwave spectroscopy. The work is very interesting and useful for a large number of journal readers. The strategy and the results are well presented and discussed, conclusions sound good and the bibliography is accurate. A nice work that merit the publication in the present form.

Reviewer: 2

Comments to the Author

The manuscript presents a clever combination of electronic structure and nuclear dynamics approximations (dubbed PCS) which leads, without the introduction of further fitting parameters, to a successful prediction of equilibrium rotational constants for a set of biomolecules, when compared to semiempirically extrapolated values from experimental data fitted to model hamiltonians.

"Parameter-free model" and "without any empirical parameter" are somewhat misleading categories for a DFT-based approach like B3LYP which by itself is empirically refined to a substantial extent. The authors of the PCS-underlying rDSD approach speak of minimally empirical models, which I consider much more adequate wording.

Also, the statement that quantum chemistry approaches rival experiment for small semi-rigid molecules is a bit optimistic in the context relevant to this manuscript, namely rotational spectroscopy. The extremely large number of significant digits in the measured frequencies is very hard to match by theory even for small, stiff polyatomic molecules - certainly not within non-relativistic Born-Oppenheimer approximations. Perhaps the author means "equilibrium" structure as the meeting point between theory and experiment, rather than the primary experimental observables such as transition frequencies. This should then be made very explicit, otherwise the reader is misled. Indeed, later on, the error target is formulated as 0.1%, which is impressive, but orders of magnitude less accurate than experimental transition frequencies in the microwave range.

I thus think that the above-mentioned statements are unneccessary exaggerations given the impressive performance and usefulness of the proposed black-box method.They may clearly mislead the more general readership of JPC Lett.

The immediate significance of this work is that it provides substantially better and less expensive theoretical reference values for experimental rotational constants of medium-sized molecules, even if they involve some non-rigidity. This is also beneficial for high level conformational rankings based on single-point energies. Whether it also applies (in attenuated form) to molecular complexes remains to be seen. There, it should also be explained why D3 dispersion correction is used rather than the recommended D4 variant.

In summary, if the misleading introductory wording is avoided and the manuscript is adopted better to the broad readership by adding some missing definitions (see below), I consider the manuscript suitable for JPC Lett.

Some technical suggestions:

small typographical errors are noted: accorate, triptophan, nucleobasis, meet/met

TMA should be defined as a full acronym.

In Table 1 the unit of the reported quantities is missing. VB_i should be explained, it is not named this way in other parts of the manuscript.

In Figure 3 DVib should be explained.

Author's Response to Peer Review Comments:

Pisa, 12 June 2023

Journal: The Journal of Physical Chemistry Letters
Manuscript ID: jz-2023-013804
Original Submission Date: 19-May-2023
Title: "Molecular Structures with Spectroscopic Accuracy for the Bricks of Life by a New Parameter-Free Model"
Author(s): Barone, Vincenzo

Dear dr. Editor,
thank you for your kind mail of June 7. In the following pages I provide a detailed answer to all the questions raised by yourself and the referees, together with an indication of the modifications performed in the revised manuscript.
I thank you and the referees for your useful suggestions and hope that now the manuscript is suitable for publication on the Journal of Physical Chemistry Letters.
Sincerely

Vincenzo Barone

Reviewer: 1

Recommendation: This paper represents a significant new contribution and should be published as is.
Author answer: I thank the referee for his/her positive judgement

Reviewer: 2

Recommendation: This paper is publishable subject to minor revisions noted. Further review is not needed.

Comments:
The manuscript presents a clever combination of electronic structure and nuclear dynamics approximations (dubbed PCS) which leads, without the introduction of further fitting parameters, to a successful prediction of equilibrium rotational constants for a set of biomolecules, when compared to semiempirically extrapolated values from experimental data fitted to model hamiltonians.

"Parameter-free model" and "without any empirical parameter" are somewhat misleading categories for a DFT-based approach like B3LYP which by itself is empirically refined to a substantial extent. The authors of the PCS-underlying rDSD approach speak of minimally empirical models, which I consider much more adequate wording.
Author answer: the referee is right, since the correct statement is that there are no empirical parameters in addition to those possibly present in the underlying electronic structure method. This is now clearly stated in the abstract, whereas all the other mentions of ‘parameter-free’ and ‘without any empirical parameter’ have been removed. The title has been modified with the same aim and now reads “Accuracy Meets Feasibility for the Structures and Rotational Constants of the Molecular Bricks of Life: a Joint Venture of DFT and Wave-Function Methods”.

Also, the statement that quantum chemistry approaches rival experiment for small semi-rigid molecules is a bit optimistic in the context relevant to this manuscript, namely rotational spectroscopy. The extremely large number of significant digits in the measured frequencies is very hard to match by theory even for small, stiff polyatomic molecules - certainly not within non-relativistic Born-Oppenheimer approximations. Perhaps the author means "equilibrium" structure as the meeting point between theory and experiment, rather than the primary experimental observables such as transition frequencies. This should then be made very explicit, otherwise the reader is misled. Indeed, later on, the error target is formulated as 0.1%, which is impressive, but orders of magnitude less accurate than experimental transition frequencies in the microwave range.
Author answer: the referee is in principle right, but the situation is a bit more complicated. Which is the experimental outcome? Any fitting of the measured frequencies implies a model Hamiltonian with the consequent intrinsic increase of the error irrespective of the comparison with QC computations. Furthermore, interpretation in structural terms for medium-sized molecules is always performed in the framework of the Born-Oppenheimer approximation and of vibrational perturbation theory. Therefore, the real error of experimental data (even when vibrational corrections come from experiment) when coming to geometrical parameters is presumably not much lower than 0.1%. In any case, since this discussion is outside the scope of the paper, the statement that quantum chemistry approaches rival experiment for small semi-rigid molecules has been removed.

I thus think that the above-mentioned statements are unneccessary exaggerations given the impressive performance and usefulness of the proposed black-box method. They may clearly mislead the more general readership of JPC Lett.
Author answer: as already mentioned all the above-mentioned statements have been removed

The immediate significance of this work is that it provides substantially better and less expensive theoretical reference values for experimental rotational constants of medium-sized molecules, even if they involve some non-rigidity. This is also beneficial for high level conformational rankings based on single-point energies. Whether it also applies (in attenuated form) to molecular complexes remains to be seen. There, it should also be explained why D3 dispersion correction is used rather than the recommended D4 variant.
Author answer: the reasons for using the D3BJ version of the rDSD functional in place of the D4 one (both versions are given in the original paper) are the negligible difference between D3BJ and D4 for intra-molecular geometrical parameters and the availability of analytical Hessians only for the D3BJ model. This is now explained on page 5.

In summary, if the misleading introductory wording is avoided and the manuscript is adopted better to the broad readership by adding some missing definitions (see below), I consider the manuscript suitable for JPC Lett.

Some technical suggestions:

small typographical errors are noted: accorate, triptophan, nucleobasis, meet/met
Author answer: all the typographical errors have been corrected

TMA should be defined as a full acronym.
Author answer: the TMA acronym is no longer used

In Table 1 the unit of the reported quantities is missing. VB_i should be explained, it is not named this way in other parts of the manuscript.
Author answer: in Table 1 the units are now given and all the acronyms have been explained

In Figure 3 DVib should be explained.

Author answer: Figure 3 has been redrawn and now DVib does not appear

Non-scientific changes:

1) The TOC has been resized and put in the same page as the abstract
2) The references now adhere to the JPC Lett. style
